# Disruption of Endothelial Cell Homeostasis Plays a Key Role in the Early Pathogenesis of Coronary Artery Abnormalities in Kawasaki Disease

**DOI:** 10.1038/srep43719

**Published:** 2017-03-03

**Authors:** Kentaro Ueno, Yumiko Ninomiya, Daisuke Hazeki, Kiminori Masuda, Yuichi Nomura, Yoshifumi Kawano

**Affiliations:** 1 Department of Pediatrics, Kagoshima University Graduate School of Medical and Dental Sciences, Kagoshima, Japan; 2 Department of Pediatrics, Kagoshima City Hospital, Kagoshima Japan

## Abstract

Disruption of endothelial cell homeostasis may be associated with the pathogenesis of coronary artery abnormalities (CAA) in Kawasaki disease (KD). We sought to clarify the poorly understood pathogenic role of endothelial cell survival and death in KD vasculitis. Human umbilical vein endothelial cells (HUVECs) stimulated with sera from KD patients, compared with sera from patients with bacterial infections, exhibited significant increases in cytotoxicity, high mobility group box protein 1 (HMGB-1), and caspase-3/7 and a decrease in phosphorylated Akt/Akt (pAkt/Akt) ratios. HUVECs stimulated with sera from KD patients treated with immunoglobulin (IG) showed significantly decreased cytotoxicity, HMGB-1, and caspase-3/7 levels and increased pAkt/Akt ratios, as compared with results for untreated HUVECs (*P* < 0.001, *P* = 0.008, *P* = 0.040, and *P* < 0.001, respectively). In HUVECs stimulated with sera from KD patients, the increased cytotoxicity levels and the suppression of increased pAkt/Akt ratios after subsequent IG treatment were closely related to the development of CAA (*P* = 0.002 and *P* = 0.035). Our data reveal that shifting the balance toward cell death rather than survival appears to perturb endothelial cell homeostasis and is closely related to the development of CAA. The cytoprotective effects of IG treatment appear to ameliorate endothelial cell homeostasis.

Kawasaki disease (KD) is an acute systemic vasculitis of unknown etiology that commonly occurs in children and is a leading cause of acquired heart disease among children in developed countries^1^,^2^. The acute vasculitis associated with KD may lead to the development of a complex set of coronary artery abnormalities (CAA)^3^. Treatment with high-dose intravenous immunoglobulin (IVIG) and aspirin effectively resolves inflammation and reduces the occurrence of CAA in KD patients^4^. However, approximately 10% to 20% of KD patients have persistent or recurrent fever after treatment with IVIG and aspirin, and these patients appear to have a high risk of developing CAA^5^,^6^. Recently, studies have shown that use of prednisolone (PSL) with IVIG and aspirin as primary treatment has a significant advantage over IVIG alone in the prevention of CAA, thus reducing the need for additional rescue treatments^7^,^8^.

Early pathology studies of fatal cases of KD have revealed that initial neutrophil infiltration of the coronary arteries, with necrosis of the arteries beginning at the luminal endothelium, occurs in the first 1–2 weeks of illness^9^,^10^. Subsequently, large saccular aneurysms are formed, which may then rupture or gradually fill with layers of thrombus^9^,^10^. Substantial intimal thickening caused by proliferation of smooth muscle cells and accumulation of fibrous tissue and matrix products were observed with a disrupted lamina interna, thus potentially resulting in progressive arterial stenosis with or without thrombosis in the late phase of KD^10^,^11^.

Generally, the pathogenesis of vascular disease is believed to involve lesion formation and remodeling, which is predominantly dictated by a balance between cell survival and death^12^. Thus, we hypothesized that the mechanism underlying the pathogenesis of KD vasculitis is ultimately an imbalance between cell survival and death triggered by cellular stress during the early stages of the disease. Here, we focused on the pathogenesis of early KD vasculitis by examining the balance between endothelial cell survival and death, using serum activity from KD patients to stimulate *in vitro* human umbilical vein endothelial cells (HUVECs), and analyzed how these processes are involved in the development of CAA. Furthermore, we investigated the therapeutic effects of immunoglobulin (IG) and PSL on HUVECs stimulated with sera from KD patients.

## Results

### Clinical Characteristics and Laboratory Findings in KD Patients and Patients with Bacterial Infections

During the study period, 42 KD patients (median, 1.7 years, IQR 0.8–2.9) and 10 patients with bacterial infections (median 1.4 years, IQR 0.9–2.1) were evaluated. The clinical characteristics and laboratory findings of both groups are shown in Table 1. Five KD patients who fulfilled the risk score criterion were treated with IVIG plus PSL^13^. No significant differences were observed with respect to sex or laboratory data, with the exception of sodium levels. Baseline white blood cell counts and C-reactive protein levels did not differ between groups.

CAA complications were observed in 8 KD patients (19.0%). The clinical characteristics and laboratory findings of the KD patients with CAA and those without CAA are shown in Table 2. No significant differences were observed in age, sex, or day of fever occurrence before treatment between the two groups. However, baseline white blood cell counts, albumin levels, sodium levels and C-reactive protein levels were significantly different between the groups.

### Comparison of Cytotoxicity Assays between KD Patients and Patients with Bacterial Infections

**Figure 1:**
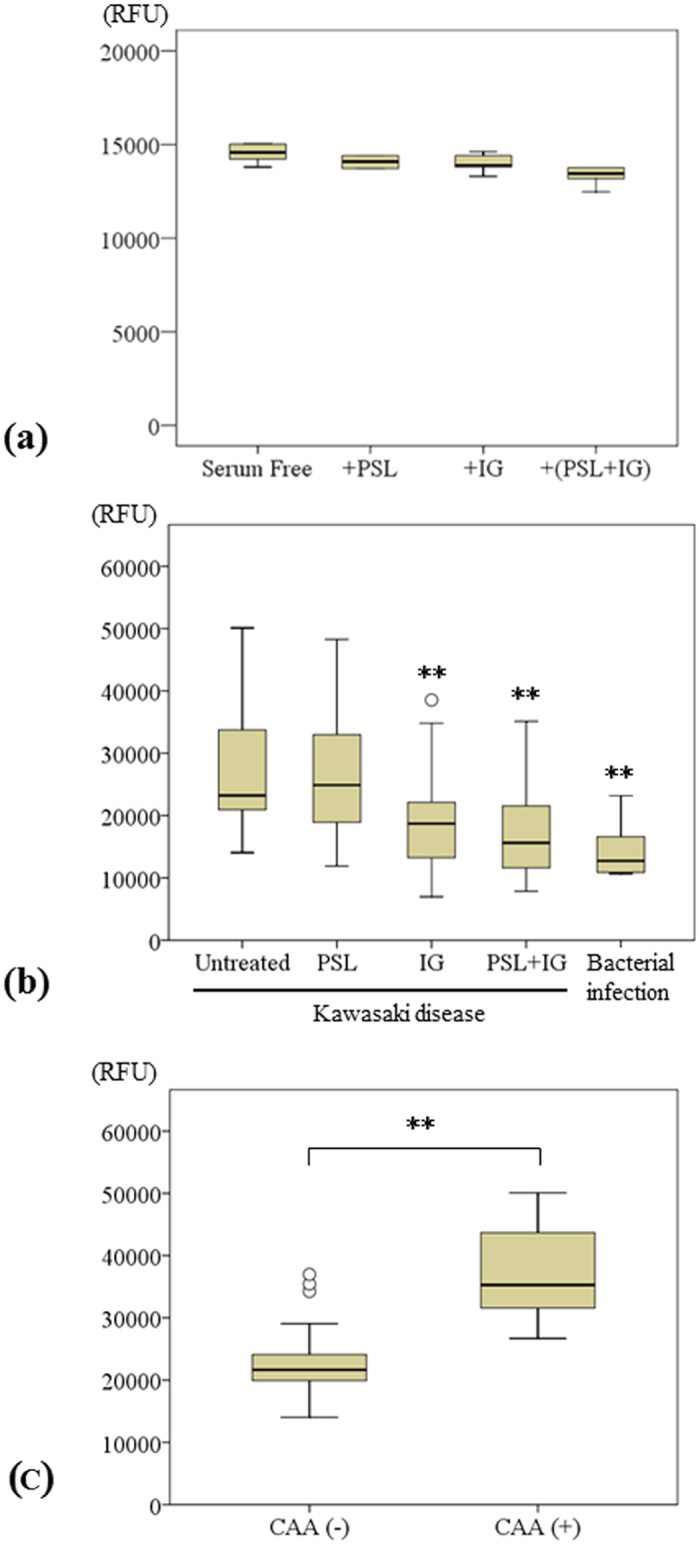
(**a**) Cytotoxicity, expressed as relative fluorescence units (RFU), was measured in serum-free HUVECs incubated with PSL, IG, and PSL + IG (median ± SD, 6 independent experiments). (**b**) RFU values were measured in HUVECs stimulated with sera from 27 Kawasaki disease (KD) patients incubated with PSL, IG, and PSL + IG and those from 6 patients with bacterial infections (median ± SD). **P < 0.01 compared with untreated. (**c**) RFU values were compared between 19 KD patients without coronary artery abnormalities (CAA) and 8 KD patients with CAA (median ± SD). **P < 0.01.

Serum-free HUVECs incubated with PSL, IG, or a combination of PSL and IG for 24 h exhibited slightly reduced cytotoxicity levels compared with those of serum-free HUVECs, but these differences were not statistically significant (n = 6, *P* = 0.375, *P* = 0.260 and *P* = 0.179, respectively) (Fig. 1a). Cytotoxicity levels in HUVECs stimulated with sera from KD patients were significantly higher than those in HUVECs stimulated with sera from patients with bacterial infections (n = 27, median 23,222 vs. n = 6, 12,734, *P* < 0.001). There was a significant decrease in cytotoxicity levels in HUVECs stimulated with sera from KD patients after incubation with IG or a combination of PSL and IG compared with the levels in untreated cells (median 18,715 vs. 15,629, *P* < 0.001, respectively) (Fig. 1b). The cytotoxicity levels were significantly higher in HUVECs treated with sera from KD patients with CAA compared with patients without CAA (n = 8, median 35,290 vs. n = 19, median 21,671, *P* = 0.002) (Fig. 1c).

### Comparison of HMGB-1 Released from Activated HUVECs between KD Patients and Patients with Bacterial Infections

**Figure 2:**
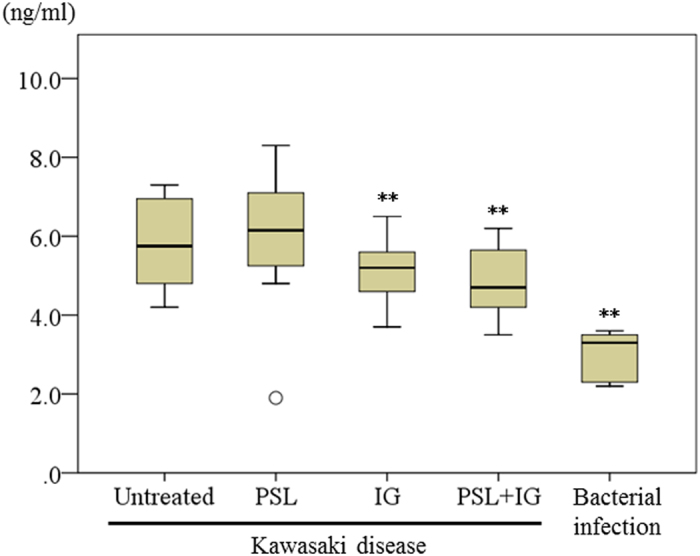
Levels of high mobility group box protein 1 in supernatants of HUVECs stimulated with sera from 16 Kawasaki disease patients and incubated with PSL, IG, and PSL + IG, and of HUVECs stimulated with sera from 6 patients with bacterial infections (median ± SD). **P < 0.01 compared with untreated.

HMGB-1 values from the culture supernatant were significantly increased in HUVECs stimulated with sera from KD patients before IVIG compared with those from bacterial infections (n = 16, median 5.8 ng/ml vs. n = 6, 3.3 ng/ml, *P* < 0.001). HUVECs stimulated with sera from KD patients after incubation with IG or a combination of PSL and IG exhibited significant reductions in HMGB-1 compared with the levels in untreated cells (median 5.2 ng/m, *P* = 0.008 vs. 4.7 ng/ml, *P* = 0.001, respectively) (Fig. 2). There was no significant differences in the values of HMGB-1 after treatment with sera from KD patients with CAA and patients without CAA (n = 6, median 6.0 ng/ml vs. n = 10, median 5.1 ng/ml, *P* = 0.410).

### Comparison of Caspase-3/7 Activity from Luminescent Cell-based Assays and Immunofluorescence Staining between KD Patients and Patients with Bacterial Infections

**Figure 3:**
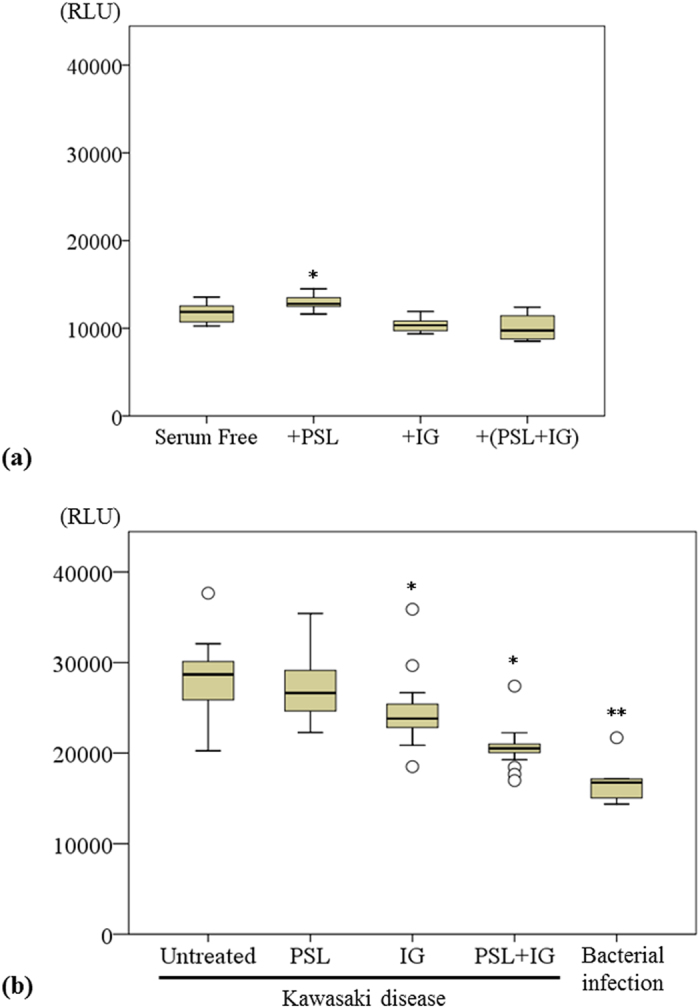
(**a**) Caspase-3/7, expressed as relative luminescence units (RLU), was measured in serum-free HUVECs incubated with PSL, IG and PSL + IG (median ± SD, 6 independent experiments). *P < 0.05 compared with serum-free HUVECs. (**b**) RLU values were measured in HUVECs stimulated with sera from 17 Kawasaki disease patients, and incubated with PSL, IG, and PSL + IG and in HUVECs stimulated with sera from 6 patients with bacterial infections (median ± SD). *P < 0.05, and **P < 0.01, compared with untreated.

Caspase-3/7 levels were higher in serum-free HUVECs incubated with PSL than in serum-free HUVECs (n = 6, *P* = 0.018). Conversely, caspase-3/7 activity in HUVECs incubated with IG and a combination of PSL and IG compared to serum-free HUVECs revealed no significant difference (n = 6, *P* = 0.168 vs. *P* = 0.074, respectively) (Fig. 3a). Caspase-3/7 levels in HUVECs stimulated with sera from KD patients were significantly higher than those from patients with bacterial infections (n = 17, median 28,688 vs. n = 6, median 16,745, *P* < 0.001). A significant decrease in caspase-3/7 levels was observed in HUVECs stimulated with sera from KD patients after incubation with IG or a combination of PSL and IG, compared with untreated HUVECs (median 23,818, *P* = 0.040; 20,520, *P* = 0.030; respectively) (Fig. 3b). No significant differences were observed in the caspase-3/7 activity between cells treated with sera from KD patients with CAA and patients without CAA (n = 5, median 29,818 vs. n = 12, median 27,702, *P* = 0.453).

**Figure 4:**
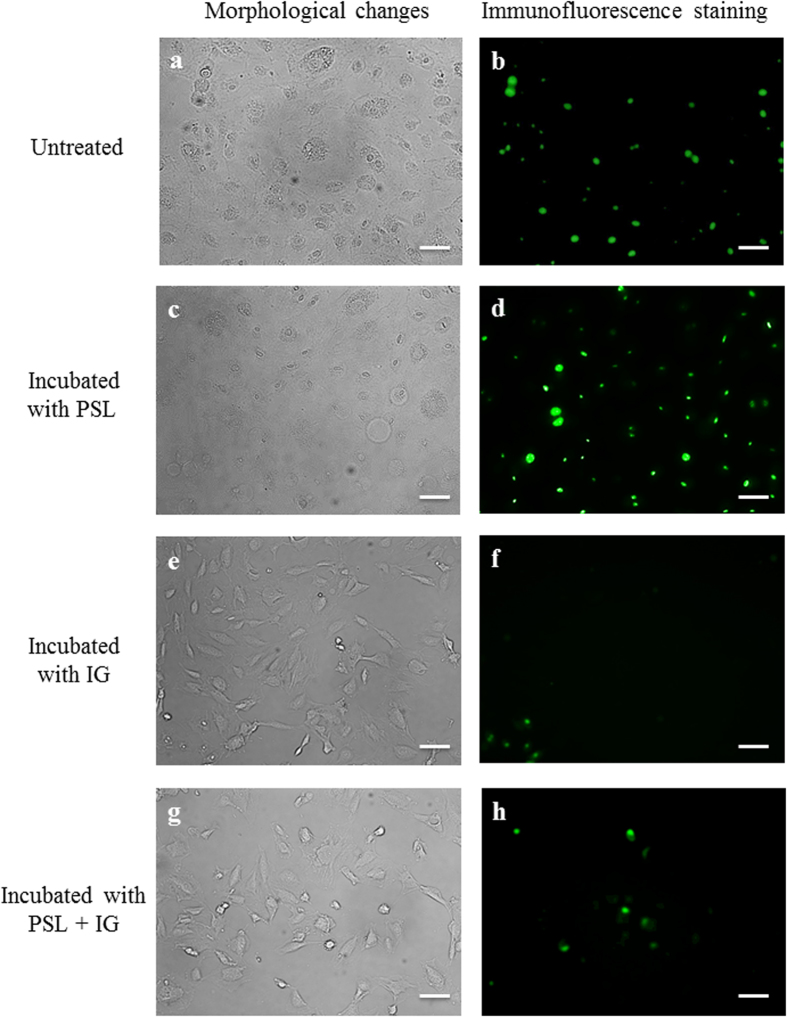
(**a–d**) Immunofluorescence staining showing morphologically condensed and fragmented nuclei (green) in HUVECs stimulated with sera from Kawasaki disease patients and those incubated with PSL. (**e–h**) Cells incubated with IG or PSL + IG maintained a morphologically normal appearance, with reduced caspase 3/7 activity. Scale bar = 100 μm.

Immunofluorescence staining exhibited morphologically condensed and fragmented nuclei visualized in fluorescent green in HUVECs stimulated with sera from KD patients; such staining patterns were observed in both untreated and PSL-treated groups. HUVECs stimulated with sera from KD patients incubated with IG or a combination of PSL and IG maintained a relatively similar appearance to that of normal cells, with a reduction in caspase 3/7 activity (Fig. 4).

### Comparison of Phosphorylated Akt/Akt Ratios in Activated HUVECs in KD Patients and Patients with Bacterial Infections

**Figure 5:**
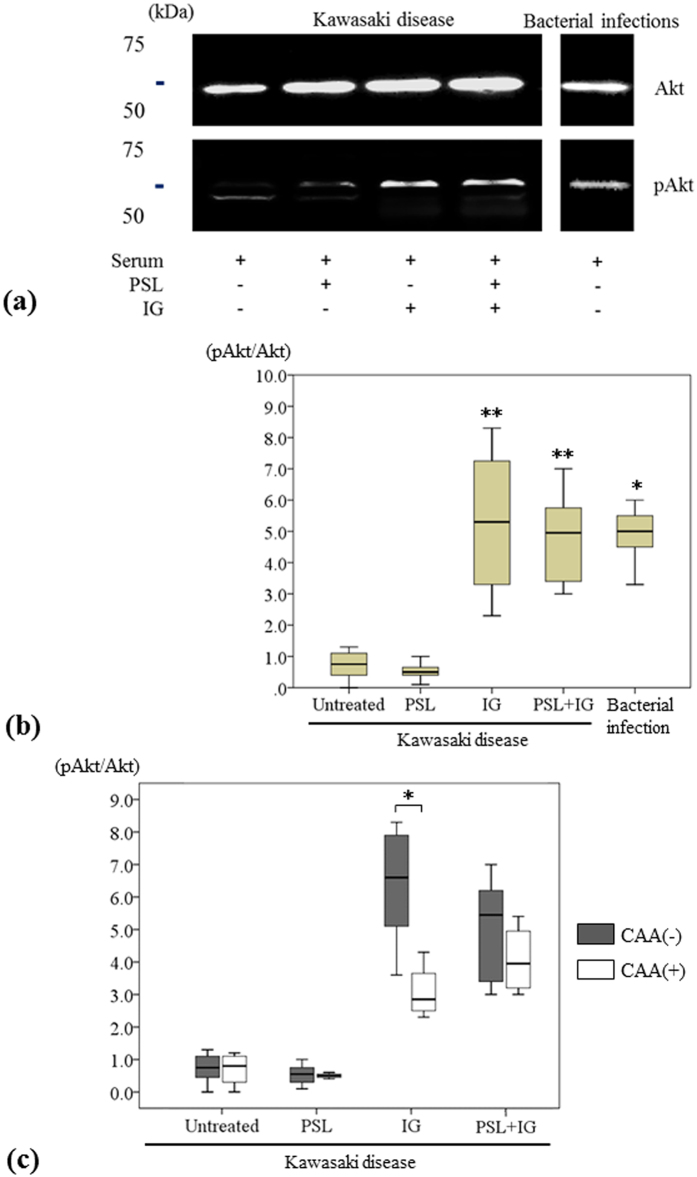
(**a**) Western blot analysis of Akt and pAkt in HUVECs stimulated with sera from Kawasaki disease (KD) patients and incubated with PSL, IG, and PSL + IG, and in HUVECs stimulated with sera from patients with bacterial infections. (**b**) Immunoblotting assay of the pAkt/Akt ratio in HUVECs stimulated with sera from 12 KD patients and incubated with PSL, IG and PSL + IG and in HUVECs stimulated with sera from 6 patients with bacterial infections (median ± SD). *P < 0.05, and **P < 0.01, compared with untreated. (**c**) The effects of PSL, IG and PSL + IG on the pAkt/Akt ratio in HUVECs stimulated with sera from 4 KD patients with CAA and 8 KD patients without CAA. *P < 0.05.

Phosphorylated Akt/Akt (pAkt/Akt) ratios were significantly lower in HUVECs stimulated with sera from KD patients than in patients with bacterial infections (n = 12, median 0.8 vs. n = 6, median 5.0, *P* = 0.004). In HUVECs stimulated with sera from KD patients, pAkt/Akt ratios after incubation with IG or a combination of PSL and IG were significantly greater than those measured in untreated HUVECs (median 5.3 vs. 5.0, *P* < 0.001, respectively) (Fig. 5a,b). Furthermore, we compared the effects of these treatments on HUVECs stimulated with sera from KD patients with respect to pAkt/Akt ratios between 4 KD patients with CAA and 8 KD patients without CAA. The differences between paired measurements of pAkt/Akt ratios on HUVECs after incubation with IG were significantly greater for cells treated with sera from KD patients without CAA compared with patients with CAA (*P* = 0.035) (Fig. 5c).

## Discussion

In this *in vitro* study, we demonstrated that increased cytotoxicity and apoptotic effects, along with decreased Akt phosphorylation, result in changes in activated endothelial cells in the presence of sera from KD patients, thus indicating that endothelial cell homeostasis is dysregulated in early stages of KD vasculitis. Additionally, increased cytotoxicity and decreased pAkt/Akt ratios were significantly associated with the development of CAA, thus suggesting that shifting the balance toward cell death rather than survival appears to be the predominant mechanism underlying coronary artery outcomes. Furthermore, IG treatments suppressed cytotoxic and apoptotic effects and induced Akt phosphorylation in endothelial cells stimulated with sera from KD patients. Our data demonstrated that the disruption of cellular homeostasis may have a crucial role in the pathogenesis of early KD vasculitis and may potentially be responsible for the development of coronary artery outcomes. IG treatment plays an important role in cytoprotection and appears to ameliorate endothelial cell homeostasis.

Cytotoxicity in endothelial cells induces various types of cell death, classified according to morphological appearance, enzymological criteria, functional aspects, or immunological characteristics^14^,^15^. Increased cytotoxicity was measured in this study on the basis of the relative quantity of dead-cell protease, a product that is rapidly released from necrotic cells. Necrosis of the arteries is believed to begin at the luminal endothelium, thus potentially resulting in progressive necrosis of the endothelium, media, and adventitia of medium-sized arteries, particularly the coronary arteries in KD^9^. Therefore, many of the key pathological features of KD are defined by necrosis, which is the product of cytotoxic effects in the early stages of disease progression. Furthermore, increased values of HMGB-1 were observed in the culture supernatants of HUVECs stimulated with sera from KD patients. HMGB-1, a ubiquitous DNA-binding protein, is passively released in considerable quantities by cells that have died in a traumatic, non-programmed manner, such as necrosis. Extracellular HMGB-1 acts as a damage-associated molecular pattern that has been reported to contribute to the pathogenesis of acute and chronic inflammatory diseases via the transduction of cellular signals. These signals induce the release of pro-inflammatory cytokines, thus leading to the maturation of dendritic cells and the chemotaxis and proliferation of smooth muscle cells^16^,^17^,^18^,^19^,^20^. However, in contrast, extracellular HMGB-1 may also promote tissue regeneration^21^. Thus, endothelial cells are damaged by cytotoxic release of extracellular HMGB-1, which may then act as an inflammatory mediator, thereby resulting in a pathological cycle that leads to persistent subacute chronic vascular inflammation in KD.

We also demonstrated that sera from KD patients induced increased caspase-3/7 activity in activated endothelial cells, as compared with sera from patients with bacterial infections. Because caspase-3 and caspase-7 are the primary executioners in apoptosis, it appears that serum components from KD patients contribute to the apoptosis of endothelial cells. Apoptosis is a programmed cell death that involves the controlled dismantling of intracellular components while avoiding inflammation and damage to surrounding cells^22^. Apoptosis plays a key role in the pathogenesis of a variety of cardiovascular diseases and is activated by multiple stressors^22^,^23^. However, endothelial cells also have complex mechanisms that protect against unintended cytotoxic or pro-apoptotic activation^25^,^26^. Thus, apoptosis may be a fundamental process for development and homeostasis. In contrast, apoptosis also contributes to a diverse range of pathological processes and is responsible for disease severity in a number of human diseases^24^. For example, in atherosclerotic plaques, apoptosis of smooth muscle cells can lead to rupture of the plaque and subsequent thrombosis^25^. Similar results have been observed in the inflamed luminal surfaces of arterial vessels in KD patients, in which an inflammatory process leads to ballooning of the vessels, disruption of laminar blood flow, and thrombus formation^10^,^26^. Simultaneously, during the process of inflammation and apoptosis, endothelial cell adherens junction proteins are degraded, thus resulting in disruption of barrier function, which may be a trigger for vascular permeability and consequent inflammation in adjacent tissues^24^. Agents facilitating increased permeability result in hypoalbuminemia and noncardiogenic edema, both of which are key features in the pathophysiology of KD. Thus, sera from KD patients induce apoptosis in vascular endothelial cells but may also contribute to the development of vascular complications in KD.

In examining the regulation of endothelial cell survival, we demonstrated that decreased Akt phosphorylation was present in HUVECs stimulated with sera from KD patients before treatment with IVIG. Akt phosphorylation may play a key role in cell survival via the phosphorylation of endothelial nitric oxide synthase (eNOS), which has both anti-apoptotic effects and contributes to the activation of cellular processes, such as cell proliferation, glucose uptake, metabolism, and angiogenesis^27^,^28^. Indeed, decreased expression of eNOS has been observed in the intimal endothelium of vessels from patients with KD vasculitis^29^. Moreover, Akt phosphorylation has physiological properties that can be cytoprotective, including the inhibition of platelet aggregation and adhesion and leukocyte activation and adhesion^30^. Akt phosphorylation is also important in mediating cell homeostasis and the integrity of the endothelium^27^,^28^. Therefore, loss of Akt phosphorylation in early stages of KD vasculitis may be responsible for disturbed vascular homeostasis and pathological conditions, thus resulting in remodeling of vascular injury sites and eventual decompensation, thereby contributing to the progression of vascular complications^31^,^32^,^33^.

Interestingly, increased cytotoxicity levels and decreased Akt phosphorylation in HUVECs stimulated with sera from KD patients were significantly associated with the development of CAA. Pathology studies in children with fatal KD have indicated that the necrosis of arteries begins early in disease progression^9^,^10^. Cytotoxic components in sera from KD patients may play a role in the pathogenesis of the disease, including the induction of neutrophil infiltration of the arterial wall^10^,^11^. Additionally, decreased eNOS expression in the intimal endothelium in patients with KD is believed to be a sign of disease progression in the histological sequence of CAA^34^. Significant increases in cytotoxicity and decreased Akt phosphorylation may shift the balance toward cell death rather than survival and are believed to be predominantly responsible for disease progression and the pathological development of CAA in KD^29^.

IG treatment plays an important role in cytoprotection and appears to ameliorate endothelial cell homeostasis by reducing cytotoxic and apoptotic effects, as well as inducing phosphorylation of Akt in HUVECs stimulated with sera from KD patients. IVIG treatment interacts with endothelial cells and consequently blockades the cytokine-induced expression of adhesion molecules, chemokines, and pro-inflammatory cytokines; these effects are believed to constitute the direct therapeutic action of IVIG in vascular and inflammatory diseases^35^. IVIG contains natural anti-cytokine antibodies, which neutralize the ‘overshoot’ of pro-inflammatory cytokines^36^, and may help to efficiently eliminate anti-endothelial antibodies that may mediate endothelial cell injury^37^. The cytoprotective effects of IVIG observed in HUVECs stimulated with sera from KD patients were not merely due to the high concentration of protein because the IVIG concentration used in this study was almost equivalent to the total serum IgG concentration 15 min after administration of 500–1,000 mg/kg of IVIG^38^. Although the subsequent cytoprotective effects of IVIG after 24 hours were not clear in this study, our results provide novel insights into the cytoprotective properties of IVIG in KD vasculitis, specifically with respect to the regulation of cell survival and death in response to vascular homeostasis.

Several debatable points regarding the effects of PSL in this study remain to be answered. First, PSL inhibits neutrophil-dependent endothelial cell injury by decreasing leukocyte emigration into inflamed sites, thus reducing cytokine-induced adhesion, and mitigating cytotoxic interactions between endothelial cells and neutrophils^39^. PSL also suppresses platelet-neutrophil aggregates, thereby indicating that it may inhibit amplified reciprocal vascular inflammatory activation in KD^40^. However, in this study, we were unable to examine the contributions of neutrophils, lymphocytes, or monocytes, owing to the serum stimulation test performed on cultured endothelial cells. Second, the anti-inflammatory effects of glucocorticoids are primarily mediated via transrepression, which requires glucocorticoid binding to glucocorticoid receptors and nuclear translocation^41^,^42^. In primary vasculitis, the anti-inflammatory effects of glucocorticoids are associated with downstream deficiencies in the glucocorticoid receptor translocation that do not affect transactivation but instead affect transrepression^42^. As glucocorticoid receptor expression has been reported to differ according to distinct cell types, and HUVECs are considered to have relatively low glucocorticoid receptor expression^42^,^43^, the relative proportion of specific glucocorticoid receptor isoforms in tissues and in cells may influence their response^44^. Additionally, we could not account for the mechanisms of IVIG unresponsiveness, because all KD patients, we analyzed in this study, were responders to single infusion of IVIG. KD patients with IVIG unresponsiveness may be influenced not only by the severe inflammation and vasculitis^13^, but also by multiple factors such as the patient’s genetic background or immunological abnormalities^45^. Thus, we were unable to demonstrate the mechanisms underlying the effects of PSL and the mechanisms of IVIG unresponsiveness in this *in vitro* model of KD vasculitis. Further studies are needed to elucidate the potential role of PSL and the mechanisms of IVIG unresponsiveness in KD vasculitis.

In conclusion, our data revealed that increased cytotoxic and apoptotic effects, as well as decreased Akt phosphorylation, were responsible for changes in activated endothelial cells in the presence of sera from KD patients that shifted the balance toward cell death rather than survival, thereby contributing to the pathogenesis of KD vasculitis. Increased cytotoxicity and suppression of Akt phosphorylation were closely related to the development of CAA. IG treatment plays an important role in cytoprotection, and appears to ameliorate endothelial cell homeostasis in KD vasculitis.

## Methods

### Patients

The study was reviewed and approved by the Kagoshima University Ethics Committee and was performed in accordance with the International Conference on Harmonisation guidelines for Good Clinical Practice and the Declaration of Helsinki. Forty two patients with KD and 10 patients with bacterial infections were included after written informed consent had been obtained from their parents. The clinical records of consecutive patients with definitive KD and bacterial infections who were referred and then admitted to Kagoshima University Graduate School of Medical and Dental Sciences and Kagoshima City Hospital from Jan 2014 to Dec 2014 were included. KD was defined on the basis of the Japanese criteria^46^. The first day of illness was defined as the first day of fever. When the medical team considered the probability of KD to be high, treatment was initiated even if the patient had not fully met the criteria for KD. All patients with KD were treated with a single infusion of IVIG at 2 g/kg. Patients also received aspirin (30 mg/kg/day), the dose of which was decreased to 3–5 mg/kg per day after they were afebrile for at least 28 days after fever onset. Patients who fulfilled the positive predictive value of the risk score developed to identify those in whom a poor initial response to IVIG was likely were treated with IVIG plus PSL at 2 mg/kg per day in three divided doses; this dose was halved every 5 days^13^. Subjects consisted of febrile patients with bacterial infections were as follows; bronchitis, pneumonia, and bacterial enterocolitis, during the same period.

### Blood Sample Selection

Serum samples were collected from KD patients and patients with bacterial infections. Serum samples were collected before the first IVIG administration in patients with KD and before initiation of antibiotics in patients with bacterial infections. Serum samples were separated through centrifugation at 700 g for 15 minutes. All samples were stored at −40 °C until the time of assay.

### Echocardiography

Two-dimensional echocardiography was performed to evaluate cardiac function and to identify whether CAA was present. Examination was performed before and after IVIG treatment at 2- to 3-day intervals during hospitalization and once a week at outpatient clinics until the end of the first month of illness. All coronary artery diameters (in mm) in KD patients were transformed into CAA z-scores by using normal Japanese coronary artery dimensions, as previously described^47^. The maximum CAA z-score among the right, left main, and left anterior descending arteries during the first month of illness was used to evaluate the presence of CAA. A maximum z-score of >2.5 was defined as the criterion for CAA.

### Endothelial Cell Culture and Preparation

Primary HUVECs (Lonza Japan, Tokyo, Japan) were obtained and cultured using an endothelial cell growth medium-2 bullet kit (Lonza, Tokyo, Japan) containing growth factors, supplements and 5% fetal bovine serum (FBS). The culture medium was changed every 24 h. When HUVECs were 70–80% confluent, the cells were trypsinized, resuspended in the culture medium and seeded into 96-, 24-, or 6-well microplates for each assay and fluorescence microscopy. HUVECs in the third passage were used for experiments.

When HUVECs were 90% confluent, the medium was exchanged with endothelial cell basal medium-2 (Lonza, Tokyo, Japan) and incubated with 7.5% serum from KD patients or patients with bacterial infections. After culturing of HUVECs for 24 h, IG (Venoglobulin-IH^TM^, Japan Blood Products Organization, Tokyo, Japan) was added at a concentration of 20 mg/ml. For those cells receiving combination therapy, PSL (Predonine, Shionogi, Osaka, Japan) was added at a concentration of 10^−6^ M per well; subsequently, wells were cultured in a 5% CO_2_ incubator for 24 h at 37 *°*C. All prepared culture media were maintained at a final pH of 7.2 to 7.4. The decision to use IG at a concentration of 20 mg/ml and PSL at concentration of 10^−6^ M was based on the concentrations used in a previous report^48^. All experiments were repeated at least three times.

### Cytotoxicity Assay

The cytotoxicity assay used was a fluorescence assay measuring the activity of dead-cell protease released from cells with impaired membrane integrity, which is associated with cell death. The cytotoxicity assay was performed according to the manufacturer’s instructions by using a MultiTox-Fluor Multiplex Cytotoxicity Assay (Promega, USA). Briefly, HUVECs were cultured at a density of 0.5 × 10^4^ cells per well in a 96-well plate in growth medium. Cells were incubated with 7.5% serum from KD patients or patients with bacterial infections in RPMI 1640 medium (Lonza, Tokyo, Japan) for a further 24 h in 96-well plates at 37 *°*C under a 5% CO_2_ atmosphere. HUVECs stimulated with sera from KD patients were treated with IG and/or PSL and incubated at 37 *°*C under a 5% CO_2_ atmosphere for a further 24 h with a final volume of 100 μl per well. Cells were incubated for 1 h with 100 μl per well of 2X MultiTox-Fluor Multiplex Cytotoxicity Assay reagent at 37 *°*C. The resultant fluorescence from dead cells was measured using a Tristar multimode microplate reader LB 941 (Berthold Technologies) at, 485 nm Ex/520 nm Em. The fluorescence produced was directly proportional to the number of dead cells. Blank values were subtracted, and the increase in activity was calculated on the basis of activity measured from untreated cells. Each sample was measured in duplicate.

### High Mobility Group Box 1 Protein Assay

Supernatant values of high mobility group box 1 protein (HMGB-1) were measured in duplicate with a commercial enzyme-linked immunosorbent assay kit (Shino-Test Corporation, Tokyo, Japan), according to the manufacturer’s instructions. The values of HMGB-1 were determined through linear regression from a standard curve derived from the HMGB-1 supplied with the kit as standard. The minimum detection value for HMGB-1 was 1.0 ng/ml.

### Caspase 3/7 Activation Assay

A luminescent caspase-3/7 activation assay was performed on each sample according to the manufacturer’s instructions by using a Caspase-Glo^®^ 3/7 assay (Promega, USA). Briefly, HUVECs were cultured at a density of 0.5 × 10^4^ cells per well in a 96-well plate in growth medium. Cells were incubated for a further 24 h with RPMI 1640 medium (Lonza, Tokyo, Japan) containing 7.5% serum from KD patients who had not yet received IVIG, in 96-well plates at 37 *°*C under a 5% CO_2_ atmosphere. HUVECs stimulated with sera from KD patients were treated with IG and/or PSL and incubated at 37 °C under a 5% CO_2_ atmosphere for a further 24 h with a final volume of 100 μl per well. Cells were transferred to a white opaque 96-well plate, after which phenol-red-free RPMI 1640 medium supplemented with 10% FBS was added. Plates were then incubated for 1 h with 100 μl per well of Caspase-Glo^®^ 3/7 reagent, and the enzymatic activity of caspase-3/7 was measured using a Tristar multimode microplate reader LB 941 (Berthold Technologies) at, 490 nm Ex/510–570 nm Em. Blank values were subtracted, and the increase in activity was calculated on the basis of activity measured from untreated cells. Each sample was measured in duplicate.

### Immunofluorescence Staining for Caspase-3/7

HUVECs were prepared on a 24-well plate and incubated with 7.5% serum in endothelial cell growth medium-2 for 24 h. HUVECs stimulated with sera from KD patients were incubated with IG and/or PSL at 37 *°*C under a 5% CO_2_ atmosphere for a further 24 h, supernatants were removed, and cells were washed with phosphate-buffered saline (PBS). The PBS was then replaced with 100 μl of phenol-red-free RPMI 1640 medium supplemented with 10% FBS. Finally, CellEvent Caspase-3/7 Green Detection Reagent assay was added to each well and incubated for 30 min at 37 *°*C under a 5% CO_2_ atmosphere, and fluorogenic substrate was used to detect activated caspase 3 and 7 (Life Technologies, Carlsbad, CA). Cells were observed using fluorescence microscopy (AXIO observer Z1; ZEISS) at 100 × magnification.

### Western Blot Analysis for Akt Phosphorylation

Third passage HUVECs were cultured at a density of 1.0 × 10^6^ cells per well in a 6-well plate with growth medium. Cells were incubated with 7.5% serum from KD patients who had not yet received IVIG, in 6-well plates at 37 *°*C under a 5% CO_2_ atmosphere for 24 h. HUVECs stimulated with sera from KD patients were incubated with IG and/or PSL at 37 °C under a 5% CO_2_ atmosphere for a further 24 h. Isolated cells were lysed and prepared for immunoblotting. Samples were subjected to a 10% gradient sodium dodecyl sulfate-polyacrylamide gel electrophoresis and were then electrotransferred to a polyvinylidene difluoride membrane. Membranes were immunoblotted using phosphorylated Akt (pAkt) at a 1:1000 dilution or Akt antibodies at a 1:2000 dilution; this was followed by incubation with secondary antibody-conjugated horseradish peroxidase (HRP) at a 1:10,000 dilution for each analysis. All blotting experiments were repeated at least twice.

### Statistical Analysis

Continuous variables are reported as median values with interquartile ranges (IQR; 25th–75th percentiles). Categorical variables are presented as frequencies and percentages. Baseline comparisons between patients were performed using Student’s t-tests, Mann–Whitney U-tests or χ^2^ analysis (with Yates’ correlation or Fisher’s exact test, as appropriate). Kruskal-Wallis tests were performed to compare data between subgroups of KD patients. All statistical analyses were performed using SPSS statistical software (version 17–0 J; SPSS Japan Inc., Tokyo, Japan). A 2-tailed *P* < 0.05 was considered statistically significance.

## Additional Information

**How to cite this article:** Ueno, K. *et al*. Disruption of Endothelial Cell Homeostasis Plays a Key Role in the Early Pathogenesis of Coronary Artery Abnormalities in Kawasaki Disease. *Sci. Rep.* **7**, 43719; doi: 10.1038/srep43719 (2017).

**Publisher's note:** Springer Nature remains neutral with regard to jurisdictional claims in published maps and institutional affiliations.

## Figures and Tables

**Table 1:** Comparison between Kawasaki disease patients and patients with bacterial infections.

Group	Kawasaki disease (n = 42)	Bacterial infections (n = 10)	*P value*
Male, N (%)	20 (47.6)	5 (50.0)	
Age at onset (years)	1.7 (0.8–2.9)	1.4 (0.9–2.1)	
Body weight (kg)	10.2 (8.4–14.3)	9.6 (8.2–11.7)	
Days of fever before treatment	4.0 (3.0–6.0)	4.0 (3.0–4.8)	
White blood cell count (×10^3^/μl)	13.5 (11.0–16.0)	14.4 (11.2–16.5)	
Neutrophil count (×10^3^/μl)	9.0 (6.8–11.3)	10.1 (9.0–13.0)	
Hematocrit (%)	33.4 (31.9–35.4)	35.2 (31.7–37.9)	
Platelet count (×10^4^/μl)	35.2 (29.0–43.3)	36.5 (33.9–43.3)	
Aspartate aminotransferase (IU/L)	39 (26–108)	35 (28–55)	
Alanine aminotransferase (IU/L)	23 (15–122)	20 (13–48)	
Lactate dehydrogenase (IU/L)	309 (268–357)	293 (237–425)	
Total protein (g/dl)	6.6 (6.2–6.9)	6.7 (5.6–7.7)	
Albumin (g/dl)	3.7 (3.4–4.0)	3.9 (3.7–3.9)	
Sodium (mEq/L)	134 (133–136)	137 (135–140)	0.039
C-reactive protein (mg/dl)	6.1 (4.3–9.3)	6.8 (5.6–8.8)	

Data are expressed as median values and interquartile range (25^th^, 75^th^ percentile), or number (proportion, %).

**Table 2:** Comparison between Kawasaki disease patients with CAA and those without CAA.

Group	Kawasaki disease with CAA (n = 8)	Kawasaki disease without CAA (n = 34)	*P value*
Male, N (%)	3 (37.5)	17 (50.0)	
Age at onset (years)	1.3 (0.7–1.8)	1.8 (0.8–3.8)	
Body weight (kg)	9.6 (8.0–11.6)	10.7 (8.4–15.7)	
Days of fever before treatment	5.0 (3.0–7.8)	4.0 (3.0–5.8)	
White blood cell count (×10^3^/μl)	14.9 (13.8–19.5)	12.7 (10.3–15.8)	0.037
Neutrophil count (×10^3^/μl)	9.9 (7.8–11.8)	8.6 (6.4–11.3)	
Hematocrit (%)	32.2 (31.1–34.2)	33.5 (32.2–35.6)	
Platelet count (×10^4^/μl)	32.7 (27.7–42.7)	36.5 (29.3–43.4)	
Aspartate aminotransferase (IU/L)	28 (21–36)	47 (27–121)	
Alanine aminotransferase (IU/L)	20 (16–51)	27 (15–174)	
Lactate dehydrogenase (IU/L)	277 (243–341)	310 (270–396)	
Total protein (g/dl)	6.7 (6.0–7.1)	6.6 (6.3–6.9)	
Albumin (g/dl)	3.4 (3.2–3.7)	3.8 (3.5–4.0)	0.038
Sodium (mEq/L)	132 (129–135)	134 (133–136)	0.049
C-reactive protein (mg/dl)	10.7 (6.1–16.7)	5.6 (3.5–8.2)	0.020

Data are expressed as median values and interquartile range (25^th^, 75^th^ percentile), or number (proportion, %).

*Abbreviations: CAA* = *coronary artery abnormalities*.
